# Histone demethylase JMJD3 protects against renal fibrosis by suppressing TGFβ and Notch signaling and preserving PTEN expression

**DOI:** 10.7150/thno.48679

**Published:** 2021-01-01

**Authors:** Chao Yu, Chongxiang Xiong, Jinhua Tang, Xiying Hou, Na Liu, George Bayliss, Shougang Zhuang

**Affiliations:** 1 Department of Nephrology, Shanghai East Hospital, Tongji University School of Medicine, Shanghai 200120, China.; 2 Department of Nephrology, the Third Affiliated Hospital of Southern Medical University, Guangzhou, 510630 China.; 3 Department of Medicine, Rhode Island Hospital and Alpert Medical School, Brown University, Providence, RI 02903.

**Keywords:** Jumonji domain containing-3, renal fibrosis, extracellular matrix, renal interstitial fibroblasts, 5/6 nephrectomy, ureteral unilateral obstruction

## Abstract

**Rationale:** The Jumonji domain containing-3 (JMJD3), a specific histone demethylase for trimethylation on histone H3 lysine 27 (H3K27me3), is associated with the pathogenesis of many diseases, but its role in renal fibrosis remains unexplored. Here we examined the role of JMJD3 and mechanisms involved in the activation of renal fibroblasts and development of renal fibrosis.

**Methods:** Murine models of 5/6 surgical nephrectomy (SNx) and ureteral unilateral obstruction (UUO) were used to assess the effect of a specific JMJD3 inhibitor, GSKJ4, and genetic deletion of JMJD3 from FOXD1 stroma-derived renal interstitial cells on the development of renal fibrosis and activation of renal interstitial fibroblasts. Cultured rat renal interstitial fibroblasts (NRK-49F) and mouse renal tubular epithelial cells (mTECs) were also used to examine JMJD3-mediated activation of profibrotic signaling.

**Results:** JMJD3 and H3K27me3 expression levels were upregulated in the kidney of mice subjected to SNx 5/6 and UUO. Pharmacological inhibition of JMJD3 with GSKJ4 or genetic deletion of JMJD3 led to worsening of renal dysfunction as well as increased deposition of extracellular matrix proteins and activation of renal interstitial fibroblasts in the injured kidney. This was coincident with decreased expression of Smad7 and enhanced expression of H3K27me3, transforming growth factor β1 (TGFβ1), Smad3, Notch1, Notch3 and Jagged1. Inhibition of JMJD3 by GSK J4 or its specific siRNA also resulted in the similar responses in cultured NRK-49F and mTECs exposed to serum or TGFβ1. Moreover, JMJD3 inhibition augmented phosphorylation of AKT and ERK1/2 *in vivo* and *in vitro*.

**Conclusion:** These results indicate that JMJD3 confers anti-fibrotic effects by limiting activation of multiple profibrotic signaling pathways and suggest that JMJD3 modulation may have therapeutic effects for chronic kidney disease.

## Introduction

Chronic kidney disease (CKD) is a common and costly global health problem with many underlying causes but few treatments as it progresses to end stage of renal disease (ESRD). Regardless of the cause of CKD, tubulointerstitial fibrosis is considered the major pathologic process that drives progression of CKD to ESRD 1. This process is dynamic and characterized by activation of renal interstitial fibroblasts and excessive accumulation of extracellular matrix (ECM) proteins, including collagens I and III, in the renal interstitium 2, 3. Despite an increasing understanding of the mechanism of renal fibrosis, there is still no available therapy that can halt the progression of CKD to ESRD. A still deeper understanding of the molecular mechanisms that mediate renal fibrosis is needed to help develop effective anti-fibrotic therapies.

In the past several decades, studies have identified many signaling pathways and factors associated with the development and progression of renal fibrosis. Among them, transforming growth factor (TGFβ)/Smad3 and Notch signaling pathways play a predominant role 2, 4. In TGFβ signaling pathway, TGFβ1 binding to TGFβ receptor II induces its activation and then recruits Smad3, resulting in its phosphorylation. Phosphorylated Smad3 is translocated to the nucleus, where it drives the expression of multiple genes associated with renal injury and fibrosis 4, 5 The Notch family includes a number of receptors and ligands. Notch 1 and Notch 3 ligand are two major receptors involved in renal fibrosis; Jagged-1 is the most studied ligand of Notch signaling 6. Upon ligand binding, the Notch receptor leads to the release of the intracellular domain of Notch (NICD), which translocates to the nucleus and activates the transcription of target genes associated with renal fibrosis 6. In addition, several other signaling pathways such as AKT and extracellular-signal-regulated kinase1/2 (ERK1/2) pathways are also activated and implicated in renal fibrosis after injury 7, 8.

Increasing evidence indicates that epigenetic modification is also involved in various renal pathological processes, including renal fibrosis 9, 10. The most studied epigenetic modifications in nephrology are DNA methylation and histone modifications 11. Histone modifications include acetylation and methylation. Histone methylation is associated with either the activation or the repression of gene transcription, depending on the specific histone residue that is modified 12. Trimethylation of histone H3 at lysine residue 27 (H3K27me3) is a histone mark associated with gene repression 12. It can be catalyzed by a methyltransferase, Zeste homolog 2 (EZH2), and reversed by a demethylase, the Jumonji domain containing-3 (JMJD3) 9, 13. JMJD3 is also called lysine-specific demethylase 6B (KDM6B) and located in the cytoplasm and nucleus; its nuclear localization is important for the demethylation of H3K27me3 and the epigenetic regulation of gene expression. JMJD3 has been identified in a variety of cellular responses, including differentiation, proliferation, senescence, and apoptosis, and linked to the pathologic processes of many diseases, such as cancer, immune diseases, and infectious diseases 13.

Recently, the functional relevance of histone methylation and demethylation in kidney disease has attracted attention. Studies by our group and others demonstrated that reduction of H3K27me3 expression levels by either pharmacological or genetic inhibition of its methyltransferase, EZH2, results in attenuation of renal fibrosis, peritoneal fibrosis, acute kidney injury and lupus, but aggravation of podocyte injury in diabetics 14-19. These findings suggest that EZH2-mediated H3K27 trimethylation can exert either a detrimental or beneficial effect on the kidney, depending on the disease type.

The functional role of JMJD3 in the pathogenesis of renal diseases remains uncertain. This study therefore aims to examine the role of JMJD3 in renal fibrosis, using two murine models of renal fibrosis - the first induced by 5/6 nephrectomy (SNx) and the second by unilateral ureteral obstruction (UUO). Our results suggest that endogenous JMJD3 is a critical epigenetic regulator that serves as a brake to minimize renal fibrosis and dysfunction in the kidney injury process.

## Results

### Pharmacological inhibition of JMJD3 aggravates renal fibrosis following ureteral obstruction

**Figure 1:**
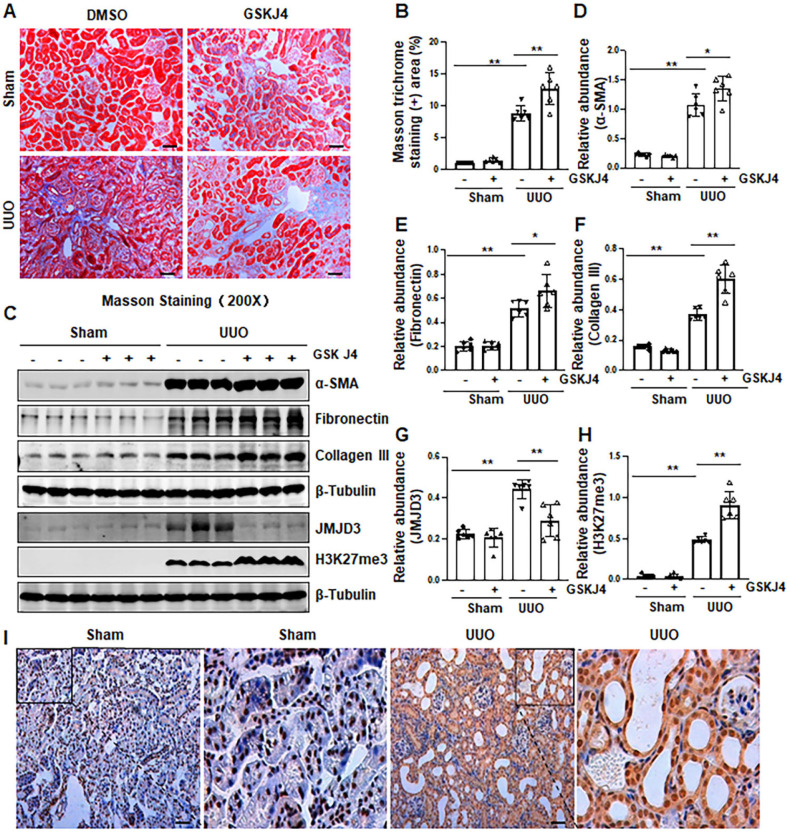
** Inhibition of JMJD3 with GSKJ4 aggravates renal fibrosis following ureteral obstruction. (A)** Photomicrographs illustrating Masson trichrome staining of kidney tissue (Original magnification ×200). **(B)** The percentage of Masson trichrome-positive tubulointerstitial area (blue) relative to the whole area was quantified. **(C)** The whole kidney tissue lysates from contralateral non-obstructed (Sham) and obstructed (UUO) were subject to immunoblot analysis with specific antibodies against α-SMA, fibronectin, collagen III, JMJD3, H3K27me3 or β-tubulin. Expression levels of α-SMA **(D)**, fibronectin **(E)**, and collagen III **(F)**, JMJD3 **(G)** and H3K27me3 **(H)** were quantified by densitometry analysis and then normalized with β-tubulin. **(I)** Photomicrographs illustrating immunohistochemical staining of JMJD3 (original magnification, ×200). Scale bar= 50 µm. Data are means ± sem.*P < 0.05; **P < 0.01 versus sham controls.

Recently, our studies demonstrated that injury to the kidney induces EZH2 expression, which is required for the trimethylation of H3K27 (H3K27me3) and renal fibrosis in a murine model of renal fibrosis induced by UUO 14. Since JMJD3 is the major demethylase of H3K27me3, and H3K27me3 is critically involved in the regulation of gene repression 9, 13, we hypothesize that JMJD3 activation would be able to antagonize the profibrotic action of EZH2 in the kidney. To test this hypothesis, we first examined the effect of GSKJ4, a specific JMJD3 inhibitor, on the development of renal fibrosis in the same UUO model used for uncovering the anti-fibrotic effect of EZH2 inhibition 14. As anticipated, administration of GSKJ4 significantly increased the deposition of ECM components, as shown by Masson trichrome staining (Figure 1A-B). In parallel, GSKJ4 treatment also enhanced UUO induced expression of α-SMA, a hallmark of activated fibroblast (myofibroblast), and fibronectin and collagen III, two ECM proteins. Notably, the expression of these proteins in control kidneys was not affected by GSKJ4 (Figure 1C-F). Along with the development of renal fibrosis following UUO injury, both JMJD3 and H3K27me3 were upregulated in the kidney with JMJD3 expression being in a time dependent manner (Figure 1C, G-H, S1A-B). Immunostaining also demonstrated the increased expression of JMJD3 in the kidney after UUO compared to that in the sham-operated kidney (Figure 1I). Co-staining of JMJD3 with α-SMA showed that JMJD3 is distributed in both renal tubular cells and interstitial fibroblasts. The overlap staining of JMJD3 and DAPI also indicates the nuclear localization of JMJD3 (Figure S2A). Administration of GSKJ4 reduced UUO-induced JMJD3 expression, and reciprocally increased H3K27me3 levels (Figure 1C, G-H). Notably, the amount of JMJD3 was minimal and H3K27me3 was barely detected in the sham-operated kidney. GSKJ4 treatment did not alter their expression levels (Figure 1C, G-H). These data indicate that JMJD3 inactivation leads to worsening renal fibrosis, in contrast to the anti-fibrotic effect of EZH2 inhibition, and suggest that injury-induced JMJD3 activation is a defensive response by the kidney to limit renal fibrosis. Furthermore, the anti-fibrotic action of JMJD3 is accompanied by demethylation of H3K27me3.

Since UTX is another demethylase of H3K27me3 9, 13, we also examined the effect of GSKJ4 on its expression in the kidney following UUO injury. A small amount of UTX protein was detected by immunoblot analysis in the sham-operated kidney, and it was slightly increased in the kidney after UUO injury. Treatment with GSKJ4 also inhibited its expression (Figure S3A-B). These data support the notion that GSKJ4 is a selective inhibitor for both JMJD3 and UTX 9, 13. Given that injury to the kidney only slightly increased UTX in the kidney compared with JMJD3, it is likely that JMJD3 predominates in mediating methylation of H3K27 and that the major effect of GSKJ4 on renal fibrosis is through inhibition of JMJD3.

### Pharmacological inhibition of JMJD3 accelerates renal dysfunction and renal fibrosis in mice with SNx

**Figure 2:**
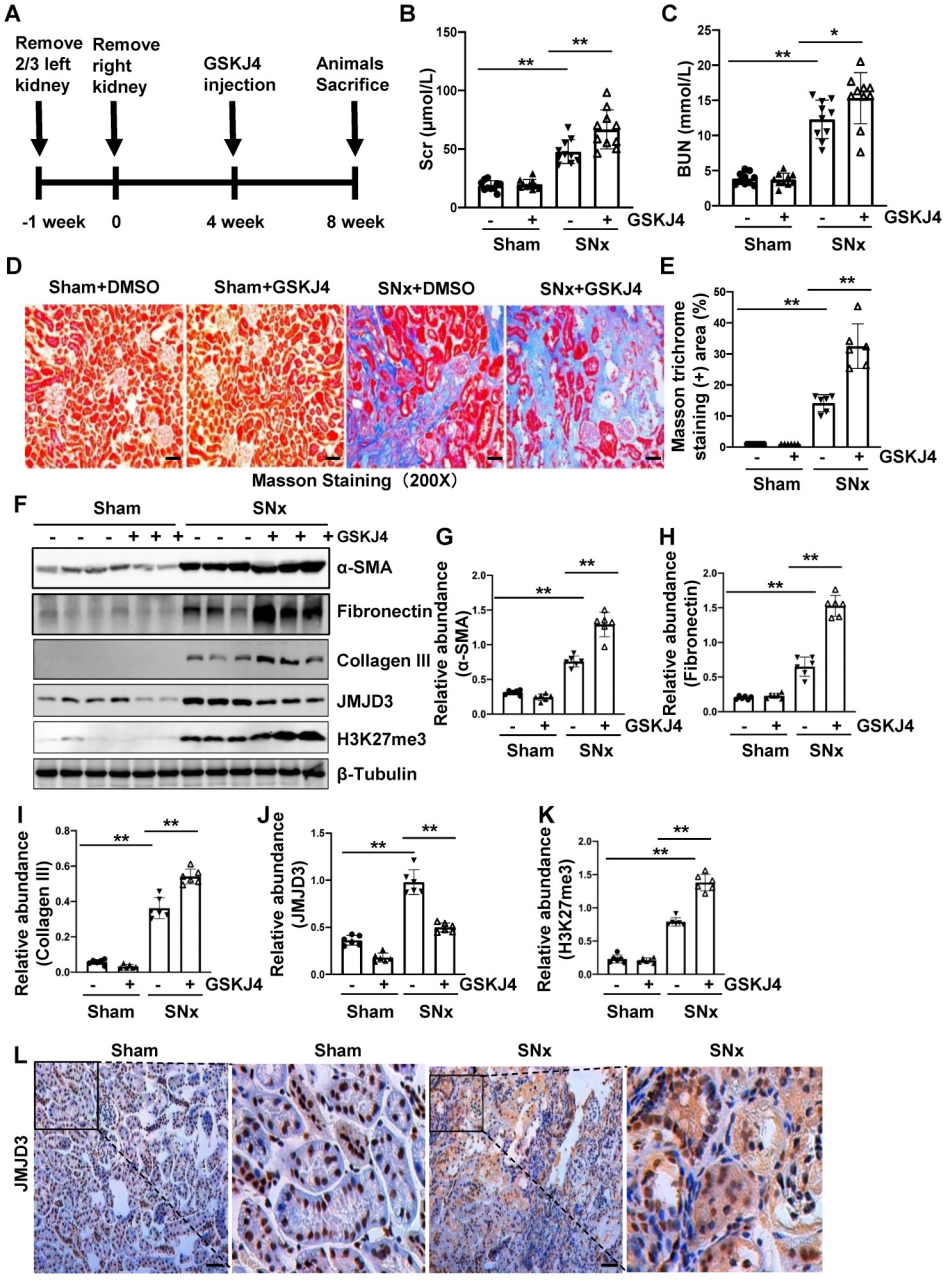
** Inhibition of JMJD3 with GSKJ4 accelerates renal dysfunction and renal fibrosis in mice with SNx. (A)** Schematic experimental design of SNx, and both blood and kidney samples were collected at 8 weeks after removal of right kidneys. **(B)** Serum creatinine levels. **(C)** Blood urea nitrogen. **(D)** Photomicrographs illustrating Masson trichrome staining of kidney tissue. Original magnification, ×200. **(E)** The percentage of Masson trichrome-positive tubulointerstitial area (blue) relative to the whole area were quantified. **(F)** Whole kidney tissue lysates from non-surgical (Sham) and remnant kidneys after surgery (SNx) were subject to immunoblot analysis with specific antibodies against α-SMA, fibronectin, collagen III, JMJD3, H3K27me3 or β-tubulin. Expression levels of α-SMA **(G)**, fibronectin **(H)**, collagen III **(I)**, JMJD3 **(J)** and H3K27me3 **(K)** were quantified by densitometry analysis and then normalized with β-tubulin. **(L)** Photomicrographs illustrating immunohistochemical staining of JMJD3 (Original magnification ×200). Scale bar= 50 µm. Data are means ± sem.*P < 0.05; **P < 0.01.

To further describe the anti-fibrotic action of JMJD3 and further demonstrate its influence on renal function, we created a murine model of SNx and then treated mice with GSKJ4 for 4 weeks, starting at 4 weeks after SNx (Figure 2A). As shown in Figure 2B-C, serum Scr and BUN levels were higher in the SNx group than in the sham-operated group. GSKJ4 administration resulted in a further worsening of renal function in the SNx group. However, this treatment did not change the blood levels of Scr and BUN in the sham-operated mice. In line with the effect of GSKJ4 on renal function, GSKJ4 also caused the expansion of fibrotic areas as indicated by Masson trichrome staining (Figure 2D-E) and induced a more pronounced expression of α-SMA, fibronectin and collagen III in the remnant kidney of mice relative to mice treated with vehicle (Figure 2F-I). Correspondingly, GSKJ4 treatment significantly reduced JMJD3, and increased H3K27me3 expression levels in SNx kidneys. The basal levels of α-SMA, fibronectin, JMJD3 and H3K27me3, but not that of collagen III, were detected in the sham operated kidney and remained the same with GSKJ4 administration (Figure 2F, J-K). Notably, SNx-induced JMJD3 expression occurred in a time dependent manner, with the maximum level at 8 weeks after injury (Figure S1C-D). Immunostaining also demonstrated that SNx increased expression in the kidney of JMJD3 (Figure 2L), which was distributed in both renal tubular cells and interstitial fibroblasts. Moreover, JMJD3 was identified in blood vessels as shown by overlapping of JMJD3 and α-SMA (Figure S2B). These data confirmed that JMJD3 activation is required to protect against renal fibrosis and further dysfunction. In line with what we had observed in UUO model, SNx also led to a slight increase of UTX in the kidney and GSKJ4 treatment inhibited this response (Figure S3C-D). Thus, the profibrotic effect of EZH2 may be counteracted by JMJD3 and UTX in the kidney injury process, with JMJD3 being predominant.

### Depletion of JMJD3 from interstitial fibroblasts aggravates renal fibrosis

**Figure 3:**
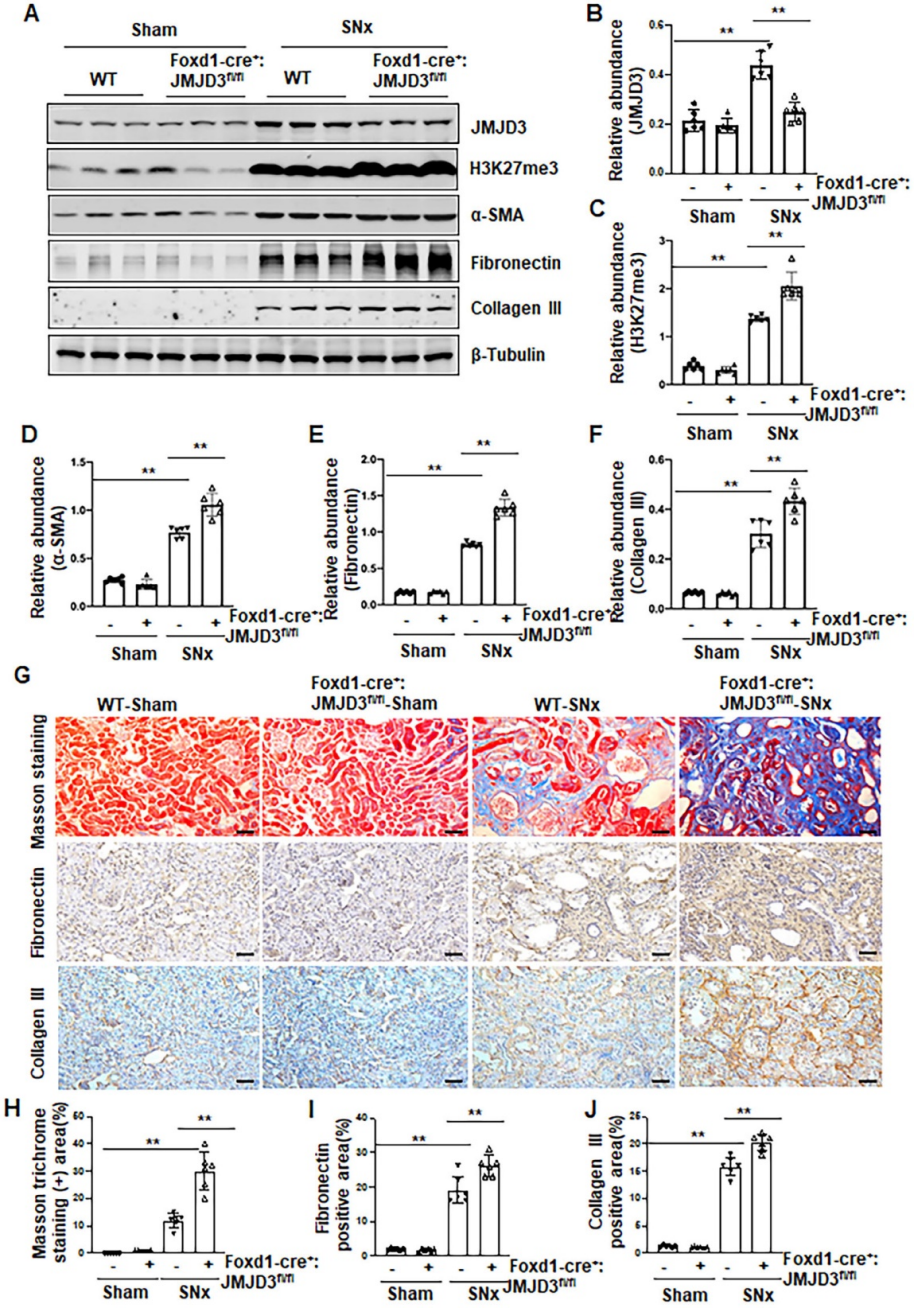
** JMJD3 depletion increases renal fibrosis after SNx.** Foxd1-cre^-^:JMJD3^fl/fl^ (JMJD3-WT) and Foxd1-cre^+^:JMJD3^fl/fl^ (JMJD3-KO) mice were left to sham operation or subjected to SNx and sacrificed 8 weeks after operations. **(A)** Whole kidney tissue lysates were subjected to immunoblot analysis with specific antibodies against JMJD3, H3K27me3, α-SMA, fibronectin, collagen III or β-tubulin. Expression levels of JMJD3 **(B)**, H3K27me3 **(C)**, α-SMA **(D)**, fibronectin **(E)**, and collagen III **(F)** were quantified by densitometry analysis and then normalized with β-tubulin. **(G)** Photomicrographs illustrating Masson trichrome staining of kidney tissue and immunohistochemical staining of fibronectin and collagen III. Scale bar= 50 µm. **(H-J)** The percentage of Masson trichrome-positive tubulointerstitial area (H) or fibronectin (I) and collagen III (J) positive areas relative to the whole area was quantified. Data are means ± sem.*P < 0.05; **P< 0.01.

Having shown that JMJD3 is expressed in the renal interstitial fibroblasts, the major source of myofibroblasts, we further investigated whether JMJD3 expressed in interstitial cells contributes to the development of renal fibrosis. During kidney development, Foxd1-expressing stromal cells give rise to cortical and medullary renal interstitial fibroblasts 20. We thus generated conditional JMJD3 depleted mice from mice with Foxd1-expressing stromal cells by breeding Foxd1-CreER^+/-^ transgenic mice with floxed JMJD3 mice. These mice developed normally. Expression levels of JMJD3 were largely reduced in the kidney of Foxd1-cre^+^:JMJD3^fl/fl^ (JMJD3-KO) mice compared to Foxd1-cre^-^:JMJD3^fl/fl^ (JMJD3-WT). SNx led to an increase in H3K27me3 as well as expression of α-SMA, fibronectin and collagen III in the kidney of WT mice, while expression of these proteins was further increased in the kidney of Foxd1-cre^+^:JMJD3^fl/fl^ (Figure 3A-F). Masson staining demonstrated that Foxd1-cre^+^:JMJD3^fl/fl^ mice developed more pronounced kidney fibrosis than WT type mice (Figure 3G-H). Immunochemical staining also showed higher expression levels of fibronectin and collagen III expression in the kidneys of Foxd1-cre^+^:JMJD3^fl/fl^ mice than in WT mice (Figure 3G, I, J). Thus, JMJD3 depleted mice subjected to SNx demonstrated increased development of renal interstitial fibrosis, suggesting that JMJD3 activation in renal fibroblasts plays an essential role in conferring an anti-fibrotic effect in the kidney following injury.

### Inhibition of JMJD3 promotes activation of renal interstitial fibroblasts in culture

**Figure 4:**
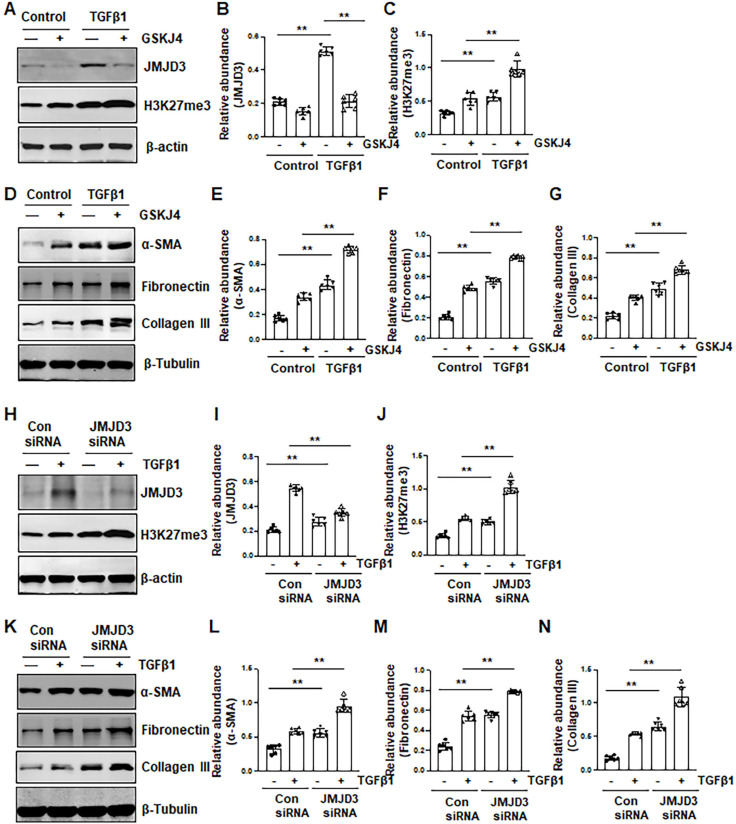
** Inhibition of JMJD3 by GSKJ4 or siRNA promotes activation of renal interstitial fibroblasts in culture exposed to TGFβ1. (A, D)** NRK-49F cells were incubated with medium containing 0.5% serum or treated with TGFβ1 (2 ng/mL) in the presence or absence of GSKJ4 (6 μM) for 36 h. Cell lysates were prepared and subject to immunoblot analysis with antibodies against JMJD3, H3K27me3, β-actin **(A)**, α-SMA, fibronectin, collagen III, β-tubulin **(D)**. Expression levels of JMJD3 **(B)**, H3K27me3 **(C)**, α-SMA **(E)**, fibronectin **(F)**, collagen III **(G)**, were quantified by densitometry analysis and then normalized with β-tubulin or β-actin, respectively. **(H, K)** NRK-49F cells were transfected control siRNA or JMJD3 siRNA and then incubated with medium containing 0.5% serum or treated with TGFβ1 (2 ng/mL) for 36 h. Cell lysates were prepared and subject to immunoblot analysis with antibodies against JMJD3, H3K27me3, β-actin **(H)** α-SMA, fibronectin, collagen III, β-tubulin **(K)**. Expression levels of JMJD3 **(I)**, H3K27me3 **(J)**, α-SMA **(L)**, fibronectin **(M)**, and collagen III **(N)** were quantified by densitometry analysis and then normalized with β-tubulin or β-actin, respectively, as indicated. Values are the means ± sem of at ≥3 independent experiments. *P < 0.05; **P < 0.01.

To verify the role of JMJD3 in the activation of renal interstitial fibroblasts, we examined the effect of GSKJ4 and silencing of JMJD3 on FBS- and TGFβ1-induced renal fibroblast activation *in vitro*. In cultured renal interstitial fibroblasts (NRK-49F) starved with 0.5% FBS, basal levels of α-SMA, fibronectin and collagen III as well as JMJD3 and H3K27me3 were observed. Exposure of cells to TGFβ1 increased their expression. Treatment with GSKJ4 reduced JMJD3 abundance, which was accompanied by increased expression of H3K27me3, α-SMA, fibronectin and collagen III (Figure 4A-G). Consistently, siRNA-mediated silencing of JMJD3 led to decreased expression of JMJD3 and reciprocally increased expression of H3K27me3 as well as α-SMA, fibronectin and collagen III (Figure 4H-N). A dose-dependent reduction of JMJD3 expression and increased expression of H3K27me3 and α-SMA, fibronectin and collagen III were also seen in this cell type treated with 5% FBS in the presence of GSKJ4, with the maximum effect at 6 μM (Figure S4A-G). Similarly, siRNA-mediated silencing of JMJD3 decreased expression of H3K27me3 and increased expression of α-SMA, fibronectin and collagen III in cultured renal fibroblasts with 5% FBS (Figure S4H-M). Therefore, inhibition of JMJD3 with either GSKJ4 or siRNA rendered renal fibroblasts more sensitive to simulation with serum and TGFβ1, leading to increased renal fibroblast activation. These data support our *in vivo* results that endogenous JMJD3 activity is required for suppression of renal interstitial fibroblast activation, while repression of JMJD3 activation potentiates transformation of renal interstitial fibroblasts to myofibroblasts.

### Pharmacological and genetic inhibition of JMJD3 enhances expression of TGFβ1, Smad3 and reduces Smad7 in the kidney following SNx

**Figure 5:**
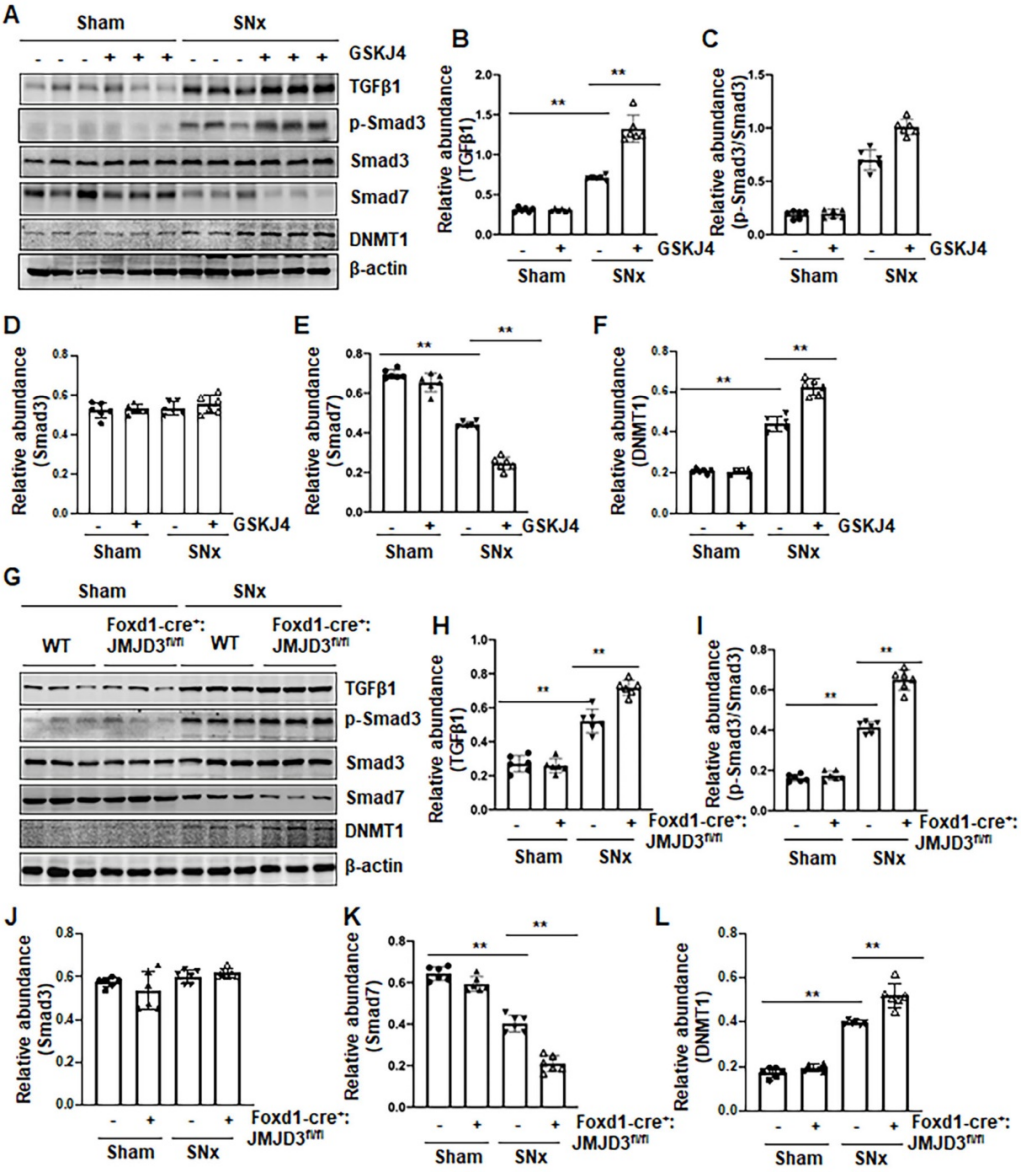
** Pharmacological and genetic inhibition of JMJD3 upregulates TGFβ1 and Smad3 and downregulates Smad7 in the kidney following SNx. (A)** The kidney tissue lysates from non-surgical (Sham) and remnant kidneys after surgery (SNx) with and without administration of GSKJ4 were subjected to immunoblot analysis with specific antibodies to TGFβ1, p-Smad3, Smad3, Smad7, DNMT1 or β-actin. Expression levels of TGFβ1 **(B)**, p-Smad3 **(C)**, Smad3 **(D)**, Smad7 **(E)**, and DNMT1 **(F)** were quantified by densitometry analysis and then normalized with β-actin. **(G)** Foxd1-cre^-^:JMJD3^fl/fl^ (JMJD3-WT) and Foxd1-cre^+^:JMJD3^fl/fl^ (JMJD3-KO) mice were left to shamed operation or subjected to SNx and sacrificed 8 weeks after operations; the whole kidney tissue lysates were subjected to immunoblot analysis with specific antibodies against TGFβ1, p-Smad3, Smad3, Smad7, DNMT1 or β-actin. Expression levels of TGFβ1, p-Smad3, Smad3, Smad7, DNMT1 or β-actin were quantified by densitometry analysis. p-Smad3 was normalized with Smad3 **(I)**; TGFβ1 **(H)**, Smad3 **(J)**, Smad7 **(K)**, DNMT1 **(L)** was normalized with β-actin. Values are the means ± sem of 6 samples. *P < 0.05; **P < 0.01.

To understand the mechanism of JMJD3-mediated anti-fibrotic effects, we first examined the effect of JMJD3 inhibition on the activation of TGFβ/Smad3 signaling in the remnant kidney after SNx injury and in cultured NRK-49F cells. As shown in Figure 5, SNx led to increased expression of TGFβ1 and phosphorylation of Smad3, and decreased expression of Smad7 in the remnant kidney, whereas either inhibition of JMJD3 by GSKJ4 or conditional depletion of JMJD3 from renal mesenchymal cells further elevated expression of TGFβ1 and p-Smad3, but reduced expression of Smad7 (Figure 5A-E, G-K). The levels of these proteins in sham-operated kidneys of mice were not affected by JMJD3 inhibition or depletion. Similar effects were also observed in cultured renal fibroblasts exposed to GSKJ4 or transfected with JMJD3 siRNA (Figure S5A-D, F-I).

Given that Smad7 is a major antagonist of the TGFβ/Smad3 signaling and that DNA methyltransferase 1 (DNMT1) is a repressor of Smad7 21, we hypothesized that JMJD3 is coupled to regulation of TGFβ/Smad3 signaling though altered DNMT1 expression. To test this hypothesis, we examined the effect of JMJD3 inhibition on DNMT1 expression in the kidney following either UUO or SNx. We demonstrated that UUO and SNx induced DNMT1 upregulation, and GSKJ4 treatment or JMJD3 depletion potentiated this response (Figure 5A, F, G, L). Similarly, inhibition of JMJD3 by GSKJ4 or JMJD3 siRNA enhanced expression levels of DNMT1 in cultured NRK-49F cells, with or without TGFβ1 exposure (Figure S5A, E, F, J).

Collectively, these data indicated that JMJD3 is an endogenous inhibitor of the TGFβ/Smad3 signaling pathway and suggest that renal protection conferred by JMJD3 is mediated, at least in part, by limiting activation of this pathway though a mechanism involved in preserving Smad7 expression. JMJD3-mediated Smad7 stabilization may occur through inhibition of DNMT1 expression.

### Pharmacological and genetic inhibition of JMJD3 increases expression of Notch1, Notch3 and Jagged-1 in the kidney following SNx and in cultured renal epithelial cells

**Figure 6:**
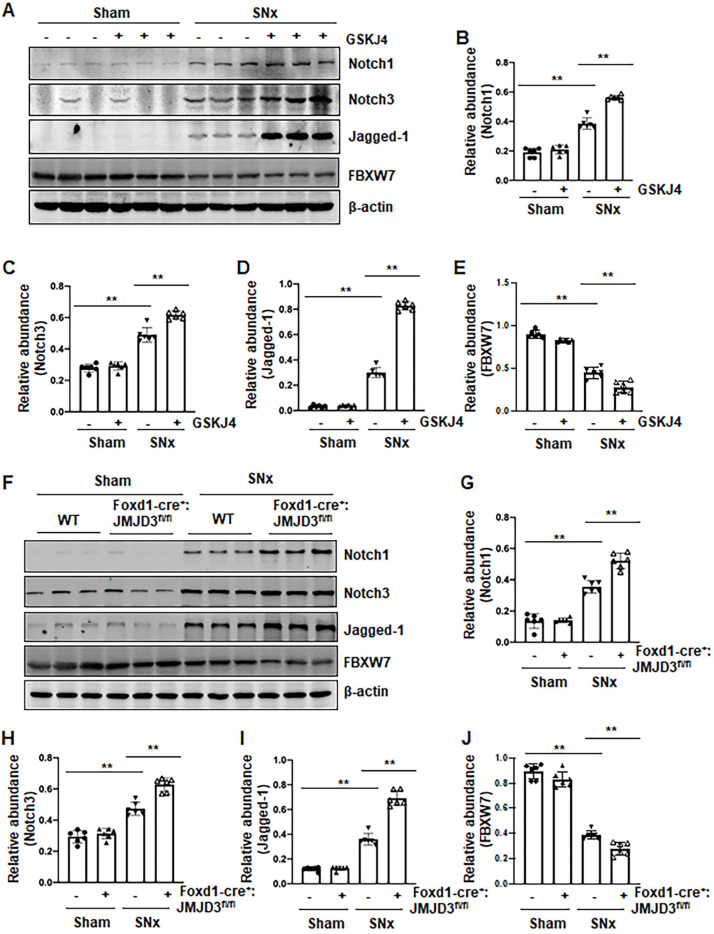
** Pharmacological and genetic inhibition of JMJD3 aggravates expression of Notch1, Notch3 and Jagged-1 in the kidney following SNx. (A)** The kidney tissue lysates from non-surgical (Sham) and remnant kidneys after surgery (SNx) were subject to immunoblot analysis with specific antibodies Notch1, Notch3, Jagged-1 or β-actin. Expression levels of Notch1 **(B)**, Notch3 **(C)**, Jagged-1 **(D)**, FBXW7 **(E)** or β-actin were quantified by densitometry analysis and then normalized with β-actin. **(F)** Foxd1-cre^-^:JMJD3^fl/fl^ (JMJD3-WT) and Foxd1-cre^+^:JMJD3^fl/fl^ (JMJD3-KO) mice were left to shamed operation or subjected to SNx and sacrificed 8 weeks after operations. The whole kidney tissue lysates were subjected to immunoblot analysis with specific antibodies against Notch1, Notch3, Jagged-1, FBXW7 or β-actin. Expression levels of Notch1 **(G)**, Notch3 **(H)**, Jagged-1 **(I)**, FBXW7 **(J)** or β-actin were quantified by densitometry analysis and normalized with β-actin. Values are the means ± sem of 6 samples. *P < 0.05; **P < 0.01.

Activation of Notch signaling is another important mechanism of renal fibrosis 6, and epithelial and interstitial Notch activation in kidneys following injury contributes to the myofibroblastic phenotype and fibrosis 22. F-box and WD repeat domain-containing 7 (FBXW7), an E3 ubiquitin ligase, functions as a negative regulator of Notch by inducing its degradation 23. To determine whether JMJD3 activity would be required for the regulation of the Notch signaling pathway, we examined the effect of JMJD3 inhibition on the expression of Notch1, Notch3, Jagged-1 and FBXW7. Compared to sham operated kidneys, Notch1, Notch3 and Jagged-1 were significantly increased, and their expression levels were elevated further by treatment with GSKJ4 in SNx remnant kidneys (Figure 6A-D). Similarly, conditional depletion of JMJD3 from mesenchymal cells enhanced SNx-induced expression of these three proteins (Figure 6F-I). Consistent with those observations *in vivo*, inhibition of JMJD3 with GSKJ4 or specific siRNA also potentiated TGFβ1-induced expression of Notch1, Notch3 and Jagged-1 in cultured mTECs (Figure S6A-D, S6F-I). In contrast, SNx led to decreased expression of FBXW7, and GSKJ4 treatment or depletion of JMJD3 from mesenchymal cells further reduced FBXW7 expression (Figure 6A, E, F, J). Similarly, TGFβ1 treatment reduced FBXW7 expression in cultured mTECs, which was potentiated further by GSKJ4 inhibition of JMJD3 or JMJD3 siRNA (Figure S6A, E, F, J). On this basis, we suggest that JMJD3 may also inhibit development of renal fibrosis by inactivation of the Notch signaling pathway through restriction of FBXW7 downregulation.

### Blocking JMJD3 with GSKJ4 enhances phosphorylation of AKT and ERK1/2 in the SNx kidney and in cultured renal fibroblasts

**Figure 7:**
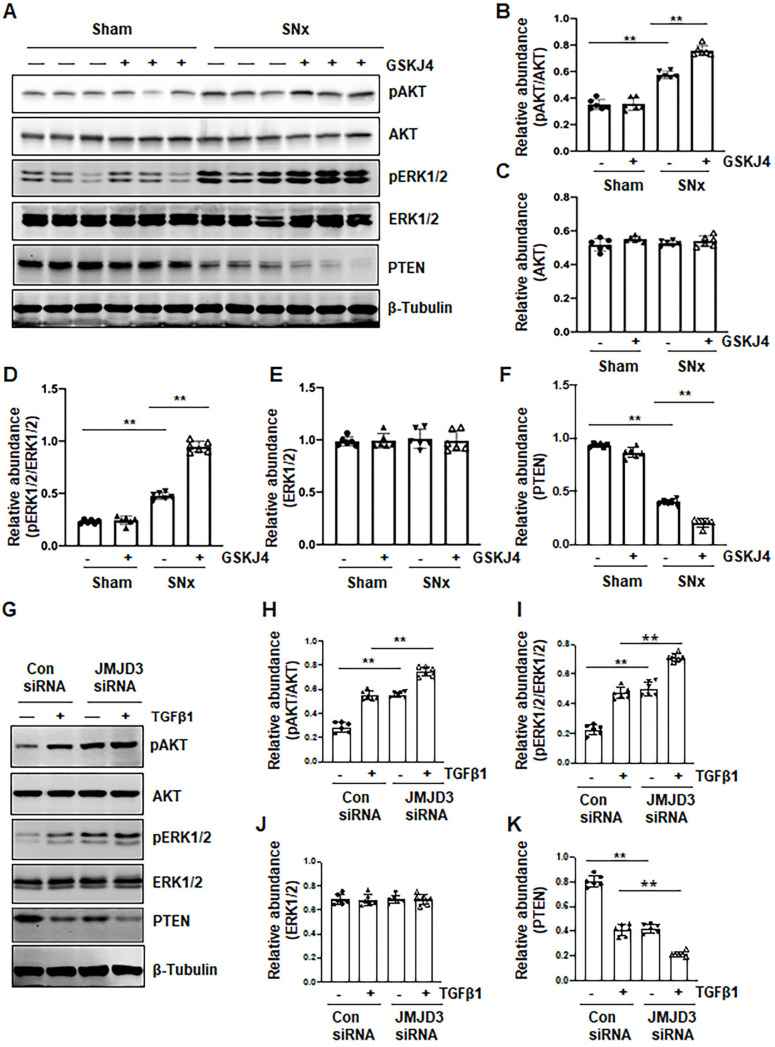
** Treatment with GSKJ4 enhances phosphorylation of AKT and ERK1/2 in the kidney after SNx and in cultured renal fibroblasts exposed to TGFβ1. (A)** The kidney tissue lysates from non-surgical (Sham) and remnant kidneys after surgery (SNx) with and without administration of GSKJ4 were subjected to immunoblot analysis with specific antibodies to pAKT, AKT, pERK1/2, ERK1/2, PTEN or β-tubulin. Expression levels of pAKT **(B)**, AKT **(C)**, pERK1/2 **(D)**, ERK1/2 **(E)**, PTEN **(F)** were quantified by densitometry analysis and then normalized with AKT, ERK1/2 or β-tubulin as indicated in figures. NRK-49F cells were transfected control siRNA or JMJD3 siRNA and then incubated with medium containing 0.5% serum or treated with TGFβ1 (2 ng/mL) for 36 h. **(G)** Cell lysates were prepared and subjected to immunoblot analysis with antibodies against pAKT, AKT, pERK1/2, ERK1/2, PTEN or β-tubulin. Expression levels of pAKT **(H)**, pERK1/2 **(I)**, ERK1/2 **(J)**, PTEN **(K)** or β-tubulin were quantified by densitometry analysis and then normalized with AKT, ERK1/2 or β-tubulin as indicated in the figure. Values are the means ± sem of at ≥3 independent experiments. *P < 0.05; **P < 0.01.

AKT and ERK1/2 signaling pathways have been reported to be involved in renal fibroblast activation and renal fibrosis 7, 8. As such, we further examined the effect of JMJD3 inhibition on the activation (phosphorylation) of these two signaling molecules *in vivo* and *in vitro*. In the sham-operated kidney, basal levels of AKT and ERK1/2 phosphorylation were observed; SNx increased their phosphorylation levels. Treatment with GSKJ4 significantly enhanced SNx-induced phosphorylation of AKT and ERK1/2 without alternating expression of their total proteins (Figure 7A-E). Similarly, basal levels of AKT and ERK1/2 phosphorylation were observed in NRK-49F cells exposed to 0.5% FBS, and increased upon TGFβ1 stimulation. Transfection of cells with JMJD3 siRNA led to enhanced phosphorylation of AKT and ERK1/2 but did not affect their total protein levels (Figure 7G-J). PTEN, a protein tyrosine phosphatase, is associated with the dephosphorylation of multiple tyrosine kinases leading to activation of AKT and ERK1/2 24. To demonstrate the possible role of JMJD3 in regulating expression of PTEN, we determined the effect of JMJD3 inhibition on PTEN expression *in vivo* and* in vitro*. As shown in Figure 7A and F, SNx reduced expression of PTEN in the injured kidney, and GSKJ4 treatment further decreased its expression. In agreement with these results, JMJD3 siRNA multiplied the effect of TGFβ1-induced down-regulation of PTEN in NRK-49F (Figure 7G and K). Collectively, these data suggest that inhibition of JMJD3 may potentiate activation of AKT and ERK1/2 signaling pathways *via* a mechanism associated with PTEN downregulation.

## Discussion

JMJD3 stimulates a wide range of genes involved in cellular responses, and its activation has been recognized as an important host response against cellular stress 13. In this study, we demonstrate that in response to fibrotic injury, JMJD3 was upregulated in the kidney, and systemic inhibition of JMJD3 with GSKJ4 or its specific depletion from stromal cells promoted renal fibroblast activation and accentuated ECM production. In cultured renal fibroblasts, either treatment with GSKJ4 or siRNA-mediated silencing of JMJD3 also aggravated these responses. Moreover, JMJD3 inhibition led to worsening of renal dysfunction and enhanced activation of several profibrotic signaling pathways, such as TGFβ/Smad3 and Jagged-1/Notch1/Notch3. Thus, our in vivo and in vitro studies support the notion that activation of JMJD3 after renal injury offers a renoprotective effect by preventing overactivation of renal interstitial fibroblasts and limiting fibrogenetic responses in the kidney injury process.

JMJD3 is a major member of JmjC histone demethylase family that specifically demethylates lysine at position 27 of histone H3 protein 13. JMJD3 exerts its biological control on gene expression by demethylating H3K27me3 13. As H3K27me3 is reciprocally regulated by JMJD3 and EZH2, the expression/activation of JMJD3 and EZH2 may determine its expression status and functional responses. Our previous and present studies have revealed that H3K27me3 as well as JMJD3 and EZH2 are all upregulated in the injured and fibrotic kidney, suggesting that both JMJD3 and EZH2 are activated and operate to regulate H3K27me3 expression. Indeed, we found that EZH2 inhibition reduced expression of H3K27me3, while JMJD3 inhibition increased its levels in the kidney following SNx and UUO, and in cultured renal fibroblasts exposed to serum and TGFβ1. Coincident with H3K27me3 alterations, we also observed a decrease of renal fibrosis with EZH2 inhibition and an increase of renal fibrosis with JMJD3 blockade. Thus, H3K27me3 seems to be functionally linked to the fibrotic process and plays an essential role in transmitting activation of JMJD3 and EZH2 to renal fibrosis. Given that JMJD3 inhibition was able to further increase H3K27 methylation and renal fibrosis, we suggest that JMJD3 expression/activation following renal injury is an adaptive response that is required to protect the kidney against renal injury and fibrosis. Supporting this hypothesis, enhanced expression of H3K27me3 and activation of renal fibroblasts were reproduced in cultured renal fibroblasts with inhibition of JMJD3 by GSKJ4 or specific JMJD3 siRNA, and in the kidney of Foxd1-cre^+^:JMJD3^fl/fl^ mice subjected to SNx. Given that Foxd1 is primarily expressed in stromal cells that give rise to cortical and medullary renal interstitial fibroblasts in adults, our findings also suggest that the impact of JMJD3 expression on renal interstitial fibroblasts is a critical step in regulating the operation of its anti-fibrotic effect. However, we cannot rule out the possibility that JMJD3 expression in renal tubular epithelial cells contributes to a defensive response to protect against partial EMT and G2/M arrest, two cellular events of renal epithelial cells that contribute to renal fibrosis 25, 26. In addition, although UTX is also a histone demethylase for H3K27me3 and sensitive to GSKJ4, its expression levels were much lower than JMJD3 in the fibrotic kidney and genetic depletion of JMJD3 showed a similar anti-fibrotic effect as GSK J4. This suggests that UTX alone may be ineffective and only work in concert with JMJD3 - a path needed for further investigation.

Our data suggest that JMJD3 mediated anti-fibrotic effects is associated with the suppression of TGFβ signaling. This was supported by our results showing that inhibition of JMJD3 increased expression of Smad3 in the SNx kidney and cultured renal fibroblasts exposed to TGFβ1 and serum. As Smad3 is a key component in TGFβ signaling responsible for driving expression of TGFβ target genes, and Smad7 blocks TGFβ1 signaling by inhibiting Smad3 phosphorylation and enhancing the degradation of TGFβ-R 27, 28, it is possible that JMJD3 mediated suppression of TGFβ1 signaling is through upregulation of Smad7. In support of this hypothesis, we observed that inhibition of JMJD3 further reduced Smad7 expression, which was coincident with increased Smad3 and TGFβ1 expression in our *in vivo* and *in vitro* models. Currently, it remains unclear how JMJD3 regulates Smad7 expression, but JMJD3 may promote its expression at the transcriptional level though DNMT1, a DNA methyltransferase 1. In this regard, a previous study showed that DNMT1 mediates repression of Smad7 and subsequent hepatic stellate cell activation and liver fibrosis 21. Our current studies also demonstrated that blocking JMJD3 with GSKJ4 resulted in increased expression of DNMT1, which was coincident with further downregulation of Smad7 *in vivo* and *in vitro*, suggesting a role for DNMT1 in mediating JMJD3 regulation of Smad7 expression. Nevertheless, whether a Smad7-independent mechanism may also contribute to JMJD3-mediated inhibition of Smad3 and TGFβ1 needs further investigation.

JMJD3 may also limit renal fibrosis and renal fibroblast activation by repressing Notch signaling. Previous studies have shown that an abnormal activation of Notch signaling promotes renal fibrosis by inducing the proliferation of renal fibroblasts and dedifferentiation of tubular epithelial cells 22, 29. In injured cells, Notch signaling is activated through the binding of the ligand Jagged to its membrane receptor proteins, Notch1 and Notch3 6. These two receptors as well as their ligand, Jagged-1, are highly expressed in the chronically injured kidney 29, 30. In this study, we demonstrated that inhibition of JMJD3 enhanced expression levels of Notch1, Notch3 and Jagged-1 in the kidney of mice subjected to SNx and UUO as well as cultured renal epithelial cells exposed to TGFβ1. This suggests that JMJD3 activation contributes to restriction of Notch signaling activation in the injured kidney. Although precise mechanisms of epigenetic regulation at Notch target genes are not yet completely understood, evidence suggests that EZH2 plays a pivotal role in the activation of Notch1 and Notch3 through H3K27me3-dependent repression of the Notch repressor FBXW7 31. We also found that SNx led to reduction of FBXW7 expression and that pharmacological and genetic inhibition of JMJD3 further reduced its expression levels in the injured kidney and TGFβ1-treated renal epithelial cells. On this basis, it is likely that activation of JMJD3, by demethylating H3K27me3, counteracts H3K27me3 dependent effects on Notch repressors, leading to inhibition of Notch signaling. In addition, Notch1 has been identified as a direct target of JMJD3 in wounded keratinocytes 32. Thus, JMJD3 may suppress Notch signaling through H3K27me3 dependent or independent mechanisms.

Although JMJD3 may regulate TGFβ1 and Notch signaling independently, it is possible that JMJD3 may also act as a regulator to connect these two signaling pathways. Emerging evidence has shown that enhanced TGFβ1 levels are associated with aberrant activation of Notch signaling. TGFβ1 can stimulate the activation of Notch1 signaling by enhancing the mRNA expression of Notch1 and Jagged-1 33, 34, whereas Notch1 signaling induces renal fibroblast activation through activation of TGFβ1/Smad3 signaling 35. On this basis, an alternative explanation for our findings is that JMJD3 inhibition-mediated upregulation of TGFβ1 and activation of Smad3 strengthens activation of Notch signaling, which in turn, further activates TGFβ1 signaling, creating a vicious circle that accelerates renal fibrosis. In this scenario, JMJD3 may regulate Notch signaling by inactivating the TGF-β1/Smad2/3 pathway cascade, and TGF-β1 may be a downstream molecule of Notch signaling.

Nevertheless, JMJD3 may protect against renal fibrosis though modulation of other signaling pathways as well. In this study, we found that JMJD3 inhibition elicited worsening fibrosis by increased phosphorylation of AKT and ERK1/2 in the kidney. Similarly, inhibition of JMJD3 promoted serum or TGFβ1-stimualted phosphorylation of these two molecules in cultured renal fibroblasts. As such, it appears that JMJD3 activation is indispensable for limiting activation of these signaling pathways. Currently, the mechanism of JMJD3 regulated phosphorylation and activation of these signaling pathways remains unknown. H3K27me3 has been reported to target and block transcriptional activation of PTEN 36, a protein phosphatase that inhibits PI3K/AKT signaling pathway 37. This is supported by our observations that inhibition of JMJD3 further decreased SNx or TGFβ1-induced downregulation of PTEN in the kidney and NRK-49F cells, respectively. Thus, JMJD3 mediated demethylation of H3K27me3 might increase PTEN expression, thereby repressing AKT phosphorylation, while inhibition of JMJD3 would lead to decreased PTEN expression, thereby enhancing AKT phosphorylation reciprocally. In addition to PTEN, JMJD3 may also act through inducing H3K27me3 demethylation to regulate other protein phosphatases associated with dephosphorylation of ERK1/2. Additional studies are required to identify the protein phosphatases regulated by JMJD3 and coupled to ERK1/2 signaling.

Although we demonstrated here that JMJD3 inhibition aggravates renal fibroblast activation and renal fibrosis in the kidney after nephrectomy and UUO injury, a recent report indicates that inhibition of JMJD3 attenuated podocyte injury and glomerular disease in models of nephrotoxicity and diabetes 38. These data, together with our findings, indicate that JMJD3 plays a dual role in kidney disease. The mechanism behind the observed discrepancy remains unclear, but may be related to cell types and disease models due to complicated regulation of JMJD3 on the target genes 13. Furthermore, demethylase-dependent and -independent effects of JMJD3 on histone methylation and its participation in different complexes may also exert context-specific effects in kidney and other diseases. Given that JMJD3 is involved in a variety of normal physiological processes and prevents renal fibrosis and renal dysfunction, complete inhibition of JMJD3 may have off-target side effects when JMJD3 is used for therapeutic targeting in patients, especially those with CKD. Conversely, induction of JMJD3 activation/expression may have a therapeutic effect on CKD. In this regard, it has been reported that JMJD3 is induced by vitamin D in colon cancer cells and is required for the protective action of vitamin D against this neoplasia 39-41. Since numerous studies have shown that vitamin D exerts an inhibitory effect on renal fibrosis 42, it is reasonable to speculate that JMJD3 may also mediate the anti-fibrotic action of vitamin D in CKD. This hypothesis is worthy of examination.

**Figure 8:**
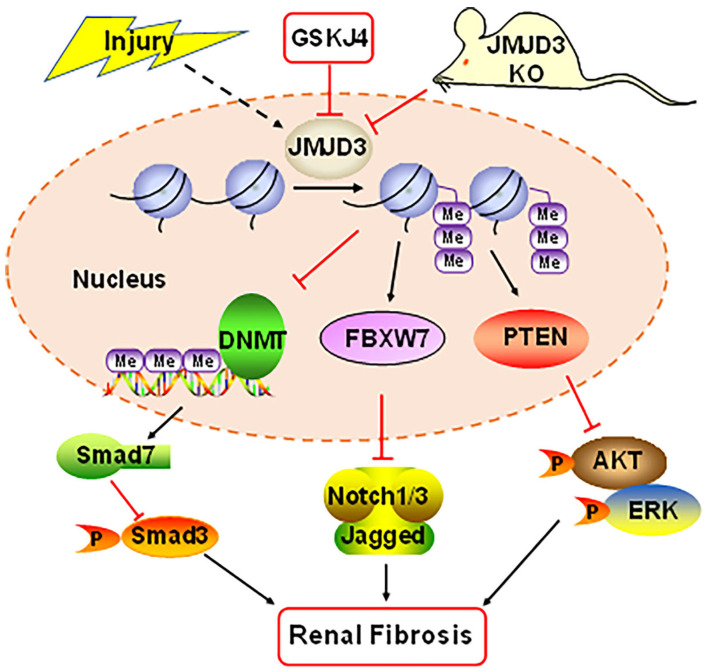
** Schematic diagram showing major findings in this study.** Fibrotic injury to the kidney increased expression and activation of JMJD3, which leads to downregulation of DNMT1 and upregulation of FBXW7 and PTEN. Decreased DNMT1 loses its inhibition on the expression of SMAD7, an antagonist of the TGFβ/Smad3 signaling; increased FBXW7 causes degradation of Notch1 and Notch3, and elevated PTEN inhibits activation of AKT and ERK1/2 signaling pathways. As a result, expression of profibrotic genes are suppressed and renal fibrogenesis is limited. Pharmacological and genetic inhibition of JMJD3 blocks all these signaling events, leading to enhanced renal fibrosis.

In summary, our study demonstrates that JMJD3 is induced in mouse models of renal fibrosis induced by UUO and SNx, and inhibition or loss of JMJD3 aggravates renal fibrosis by allowing activation of multiple profibrotic signaling pathways that otherwise would have been suppressed by JMJD3 demethylation of H3K27me3 as indicated in Figure 8. These findings suggest that renal activation of JMJD3 after chronic injury is a protective response to minimize renal fibrosis. This is in sharp contrast to the role of the H3K27me3 methyltransferase EZH2, which induces renal fibroblast activation and renal fibrosis. Considering that JMJD3 plays a dual role in many diseases, JMJD3 targeted therapy should be pursed in accordance with disease types in a clinical setting.

## Materials and Methods

### Antibodies and Agents

Antibodies to H3K27me3, JMJD3, p-Smad3, Smad3, p-AKT, AKT, p-ERK1/2, ERK1/2, Notch1, Jagged-1, UTX and DNMT1 were purchased from Cell Signaling Technology (MA, USA). Antibodies to fibronectin, α-SMA, collagen III, JMJD3, TGF-β1, Notch3 were purchased from Abcam (MA, USA). An antibody to Smad7 was obtained from Santa Cruz Biotechnology (CA, USA). An antibody to β-actin, PTEN and FBXW7 were obtained from proteintech (Wuhan, China). β-tubulin was purchased from Sigma-Aldrich (MO, USA). GSKJ4 was purchased from Selleck (Houston, USA). DMSO and other chemicals were obtained from Beyotime (Shanghai, China). Lipofectamine 3000 was obtained from Invitrogen-Thermo Fisher Scientific (CA, USA).

### Cell Culture and Treatment

Rat renal interstitial fibroblasts (NRK-49F) and murine renal tubular epithelial cells (mTECs) were cultured in DMEM with F12 containing 5% or 10% FBS. Before various treatments, cells were starved with the medium containing 0.5% FBS for 24 h. Different doses of GSKJ4 (0-6 μM) were added to the culture and then incubated for the indicated time in figure legends. To determine the role of JMJD3 in TGFβ1-induced renal fibroblast activation, TGFβ1 was added to the cultured NRK-49F and renal epithelial cells for 36 h in the presence or absence of GSKJ4.

### Transfection of siRNA

The siRNA oligonucleotides targeted to rat or mouse JMJD3 were used to downregulate JMJD3 (Shanghai, China). NRK-49F and mTECs were seeded in the antibiotic-free medium and grown followed by transfection with siRNA (100 pmol) specific for JMJD3 using Lipofectamine 3000 (CA, USA) according to the manufacturer's instructions. In parallel, scrambled siRNA (100 pmol) was used as a control for off-target changes in NRK-49F and renal epithelial cells; 6 h after transfection, the medium was changed to DMEM with F12 containing 0.5 % FBS for starve and then cells were incubated with TGFβ1 (2 ng/mL) for an additional 36 h before being harvested for analysis.

### Animals Models of Renal Fibrosis and Treatment

Male C57BL/6 mice aged 8 weeks were purchased from Shanghai Jie Si Jie Laboratory Animal Co. Ltd (Shanghai, China). JMJD3^fl/fl^ and Foxd1-Cre mice (Foxd1-CreER^+/-^) were purchased from the Jackson Laboratory (Bar Harbor, ME, USA). JMJD3 knockout mice were generated by breeding JMJD3^fl/fl^ mice with Foxd1-CreER^+/-^ to obtain Foxd1-Cre^+^:JMJD3^fl/fl^ mice (JMJD3-KO), Foxd1-Cre^-^:JMJD3^fl/fl^ mice (JMJD3-WT). The animals had free access to standard mouse chow and tap water. The animal use and care and experimental protocols were reviewed and approved by the Institutional Animal Care and Use Committee of the Tongji University School of medicine, China. The animal experiments were conducted in accordance with the National Institutes of Health Guidelines on the use of laboratory animals.

Both UUO and SNx models of renal fibrosis were used in this study. The UUO model was established as described previously 43. In brief, animals underwent laparotomy with a 1-cm dorsal incision, and the left ureter was isolated and ligated. The contralateral kidney was used as a control. SNx model was established by a 5/6 kidney resection as described previously 44, 45 except mice were used. Briefly, mice were anesthetized by the subcutaneous injection of 4% chloral hydrate at 0.1 mL/per 10g body weight followed by laparotomy and removal of 2/3 of the left kidney. One week later, the whole right kidney was removed under anesthesia. The sham and GSKJ4 treated animals underwent sham-operation but without any kidney resection.

For UUO model, C57BL/6 mice were randomly divided into 4 groups with 6 in each group: (i) sham group, (ii) GSKJ4-treated sham group, (ii) UUO group and (iv) GSKJ4-treated UUO group. Immediately after the operation, mice received the vehicle (equal volume of DMSO and saline) or GSKJ4 at daily dose of 100 mg/kg *via* I.P. for 6 days. The animals were euthanized, and the kidneys were collected at day 7 after UUO for protein analysis and histologic examination.

For SNx model, C57BL/6 mice were randomly divided into 4 groups with 10 mice in each group: (i) sham group, (ii) GSKJ4-treated sham group, (iii) SNx group and (iv) GSKJ4-treated SNx group. At 4 weeks after the operation, mice in the GSKJ4-treated sham and GSKJ4-treated SNx group were administrated GSKJ4 at daily dose of 100 mg/kg intraperitoneally for 4 weeks. Mice in the sham and the SNx alone groups were given an equal volume of DMSO and saline in the same way. At 8 weeks after operation (4 weeks after treatment), the animals were sacrificed and the blood and renal samples were collected for further analysis. The JMJD3 knockout mice were also used to establish SNx model. They were divided into 4 groups: (i) WT-Sham group, (ii) Foxd1-Cre^-^: JMJD3^fl/fl^ -Sham group, (iii) WT-SNx group and Foxd1-Cre^+^: JMJD3^fl/fl^ -SNx group. All the surgical procedures in knockout mice were the same as in C57BL/6 mice with GSKJ4 treatment as described above.

### Renal Function Analysis

Blood samples were collected from mice eyes. The serum was made by centrifugation at speed of 1500rpm for 15 min at 4 °C, and the serum Scr level was determined by Creatinine Assay Kit; the serum BUN level was detected by a BUN kit (Nanjing Jiancheng Bioengineering Institute, Nanjing, China), in accordance with the manufacturer's instructions.

### Western Blot Analysis

To prepare protein samples for western blotting, kidney tissue samples and cultured cells were homogenized in the presence of RIPA and a protease inhibitor cocktail. In brief, a total of 25 µg protein were separated by SDS-PAGE gel electrophoresis and transferred to PVDF membrane in a tank. The membrane was blocked with 5% nonfat milk for 1 hour at room temperature, and then incubated with specific primary antibodies at 4 °C overnight. After being washed three times in TBS with Tween-20, the membrane was incubated with a secondary antibody for 1 h at room temperature. Followed by TBS rinsing, bound antibodies were visualized by fluorescence detection.

### Pathological Assessment

The kidney tissues were fixed in 4% paraformaldehyde for wax block embedding. Masson trichrome was applied in kidney sections (thickness 3-5 µm) to examine the pathological changes. Deparaffinized sections were stained with Masson trichrome staining performed according to the protocol provided by the manufacturer and observed by light microscopy. Semi-quantitative assessment of Masson trichrome was performed with ImageJ to measure the deposition of ECM in the renal interstitium.

### Immunohistochemistry and Immunofluorescence staining

Immunohistochemistry and immunofluorescence staining were performed in kidney sections (thickness 3-5 µm) with various antibodies as we previously described 14. Antibodies to fibronectin, collagen III, α-SMA, JMJD3 were used. Photographs were blindly taken at random fields under a microscope ×200 optical magnification.

### Statistical analyses

Data depicted in graphs represent the means ± SEM for each group and subjected to one-way analysis of variance (ANOVA). Multiple means were compared using Tukey test, and differences between two groups were determined using Student's t test. Statistically significant difference between mean values was *P <* 0.05.

## Abbreviations

JMJD3: jumonji domain containing 3
H3K27me3: trimethylation on histone H3 lysine 27
SNx: 5/6 nephrectomy
UUO: ureteral unilateral obstruction
TGFβ1: transforming growth factor β1
CKD: chronic kidney disease
ESRD: end stage of renal disease
ECM: extracellular matrix
ERK1/2: extracellular-signal-regulated kinase1/2
KDM6B: lysine-specific demethylase 6B
NRK-49F: rat renal interstitial fibroblasts
mTECs: mouse renal epithelial cells
