# Supplementary material for: Histone demethylase JMJD3 protects against renal fibrosis by suppressing TGFβ and Notch signaling and preserving PTEN expression
Source: Theranostics. 2021 Jan 1;11(6):2706–21. doi: 10.7150/thno.48679 (PMC7806480; doi:10.7150/thno.48679)
Supplement: Supplementary file 1 — Supplementary figures and tables. [file thnov11p2706s1.pdf]

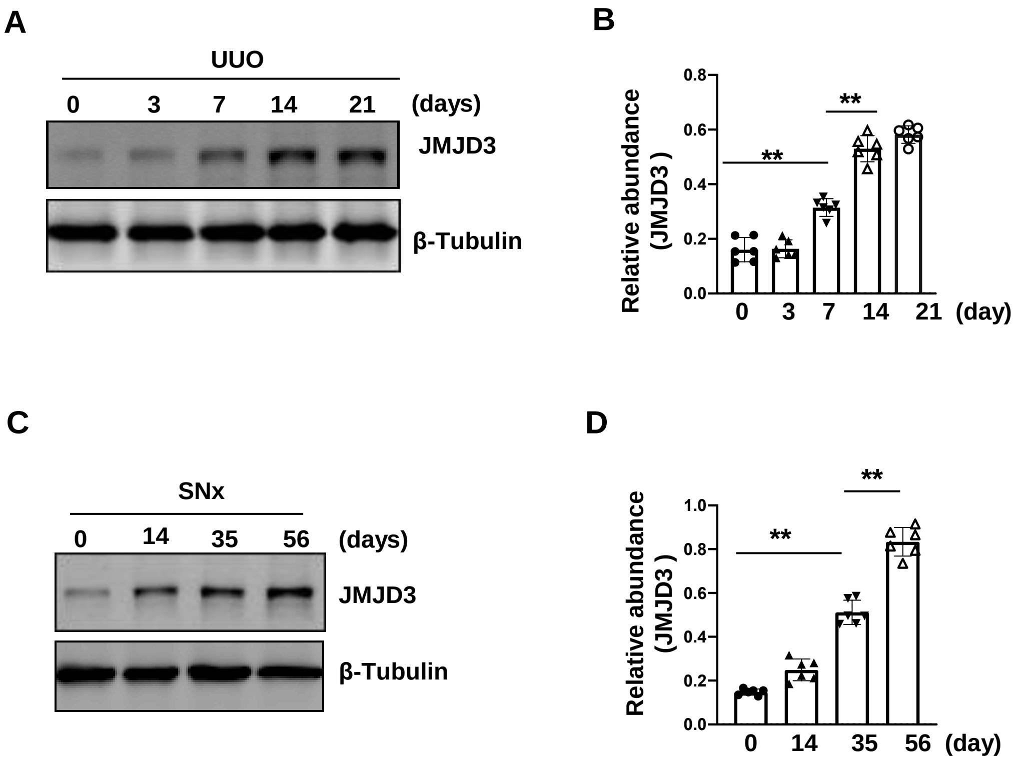

Figure S1. The time course of UUO or 5/6 nephrectomy (SNx)-induced expression of JMJD3. After UUO (A) or SNx (B), kidneys were collected at different time points as indicated. The whole kidney lysates were subject to immunoblot analysis with specific antibodies to JMJD3 or β-tubulin. Expression levels of JMJD3 and β-tubulin were quantified by densitometry analysis. (B, D) JMJD3 was normalized with β-tubulin. Values are the means ± sem of 6 samples. ***P*<0.01

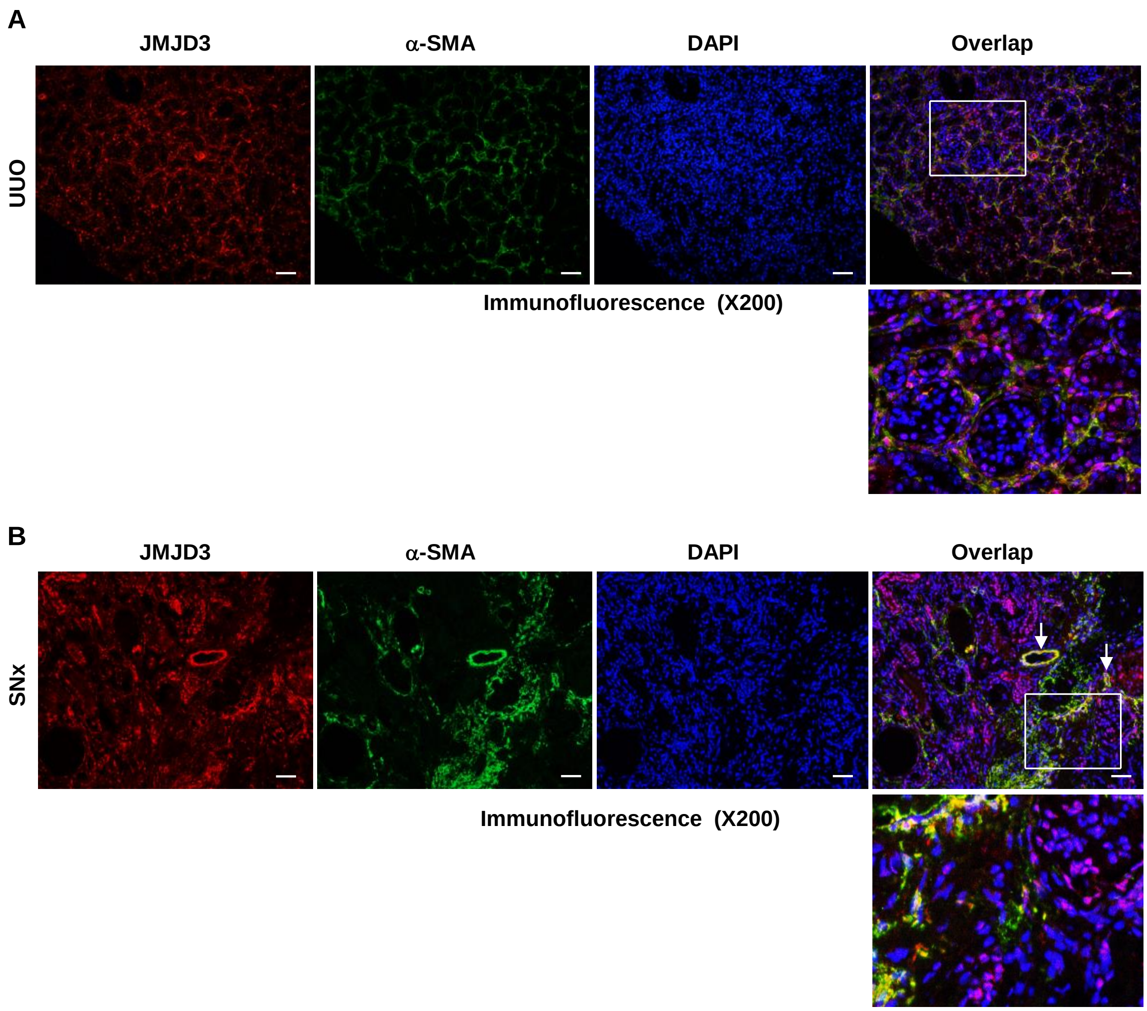

Figure S2. Expression of JMJD3 in the kidney after UUO (A) and SNx (B). The kidney was collected at 7 days after UUO or 56 days after SNx. Photomicrographs illustrating immunofluorecent co-staining of JMJD3 with α-SMA (original magnification, ×200). JMJD3 is expressed in the nucleus of both renal tubular cells and renal interstitial fibroblasts. JMJD3 was also co-stained with α-SMA (Yellow) in the cytosol of some interstitial fibroblasts and small arteries (white arrows), indicating its expression in the cytoplasm in this cell type and renal arteries. (Original magnification ×200)

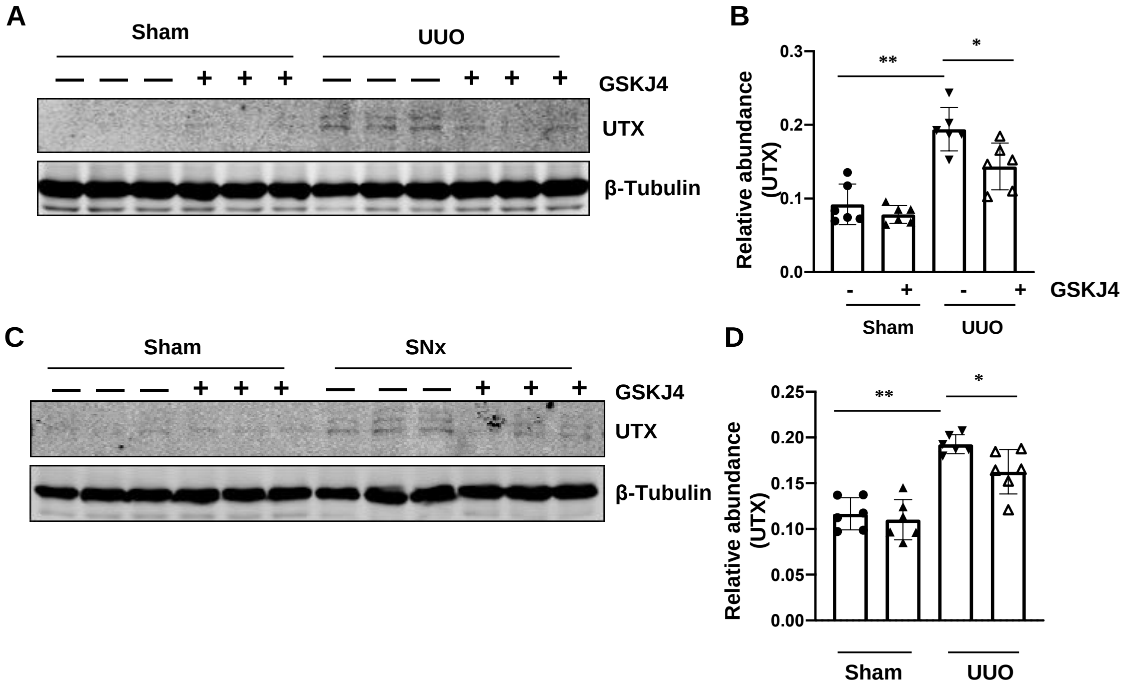

Figure S3. Effect of GSKJ4 on the expression of UTX expression in the kidney after UUO and 5/6 nephrectomy (SNx). The kidney tissue lysates from Sham-operated, UUO injured (UUO) (A) or remnant kidneys after surgery (SNx)(C) with and without administration of GSKJ4 were subjected to immunoblot analysis with specific antibodies to UTX or β-Tubulin. (B, D) Expression levels of UTX were quantified by densitometry analysis and then normalized with β-Tubuin. Values are the means ± sem of 6 samples. **P<0.01

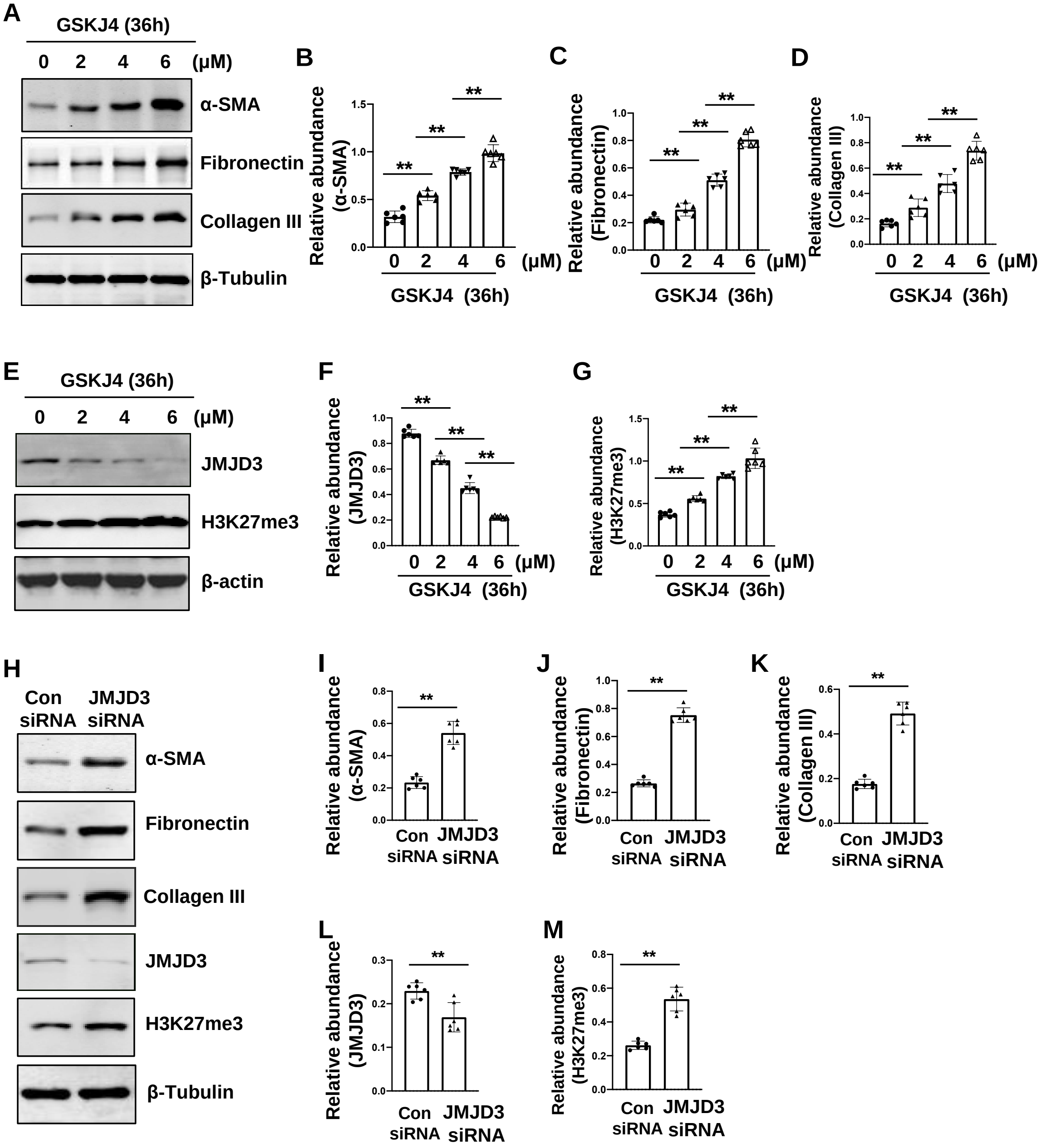

Figure S4. Inhibition of JMJD3 by GSKJ4 or siRNA promotes activation of renal interstitial fibroblasts induced by serum. (A, E) NRK-49F cells were incubated with medium containing 5 % serum in the presence or absence of GSKJ4 (0-6 μM) for 36 h. Cell lysates were prepared and subjected to immunoblot analysis with antibodies against α-SMA, fibronectin, collagen III, β-tubulin (A), JMJD3, H3K27me3, β-actin (E). Expression levels of α-SMA (B), fibronectin (C), collagen III (D), JMJD3 (F), H3K27me3 (G) were quantified by densitometry analysis and then normalized with β-tubulin or β-actin, respectively, as indicated. (H) NRK-49F cells were transfected with control siRNA or JMJD3 siRNA and then incubated with medium containing 0.5 % serum for 36 h. Cell lysates were prepared and subjected to immunoblot analysis with antibodies against α-SMA, fibronectin, collagen III, JMJD3, H3K27me3, β-tubulin. Expression levels of α-SMA (I), fibronectin (J), collagen III (K), JMJD3 (L), H3K27me3 (M), β-

tubulin were quantified by densitometry analysis and then normalized with β-tubulin. Values are the means ± sem of at ≥3 independent experiments. **P*<0.05; ***P*<0.01

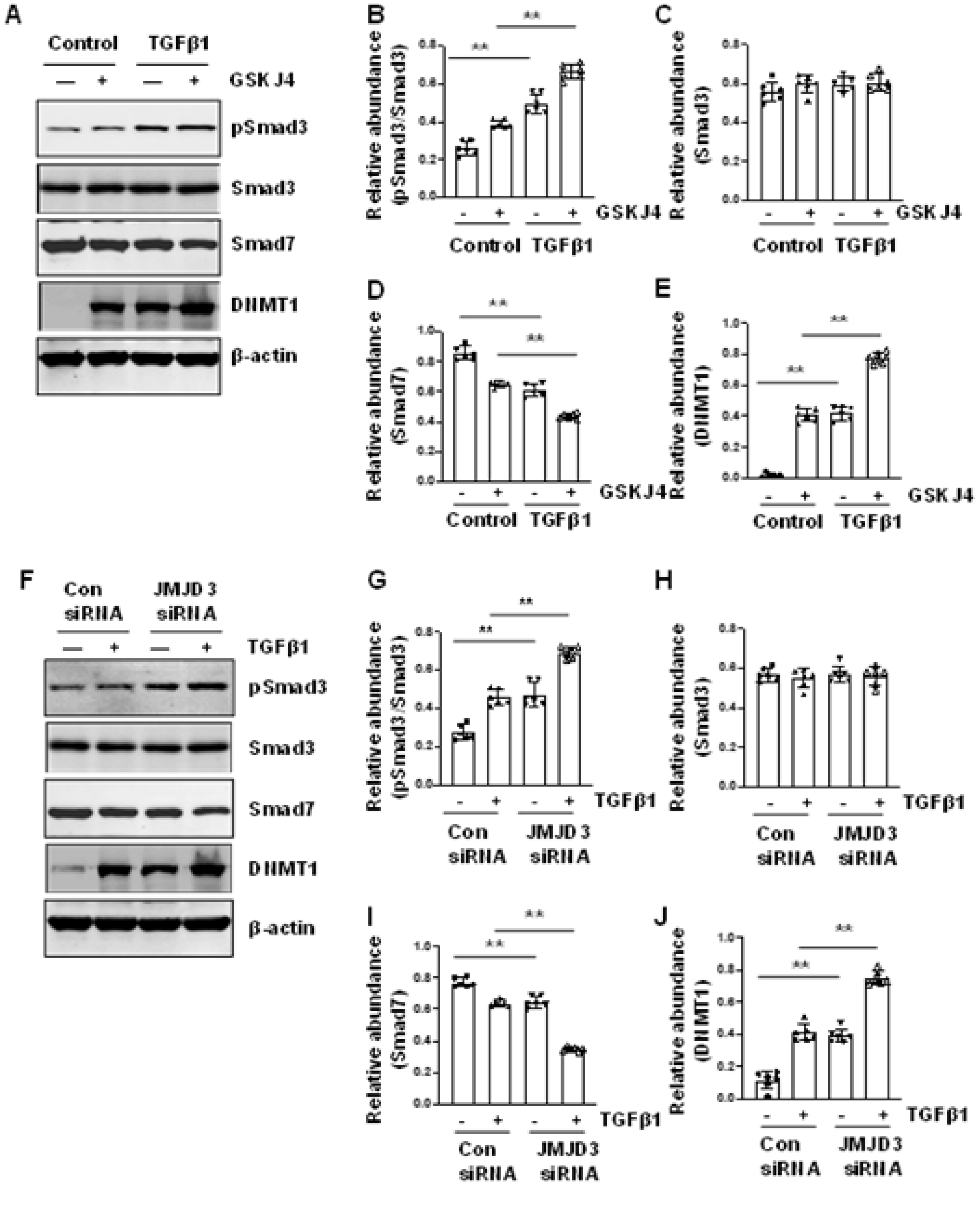

Figure S5. Inhibition of JMJD3 by GSKJ4 or siRNA promotes Smad3 phosphorylation and expression of DNMT1, as well as reduces Smad7 expression in cultured renal interstitial fibroblasts exposed to TGFβ1. (A) NRK-49F cells were incubated with medium containing 0.5% serum or treated with TGFβ1 (2 ng/ml) in the presence or absence of GSKJ4 (6 μM) for 36 h. Cell lysates were prepared and subjected to immunoblot analysis with antibodies against pSmad3, Smad3, Smad7, DNMT1, β-actin. Expression levels of pSmad3 (B), Smad3(C), Smad7 (D),

DNMT1 (E) or β-actin were quantified by densitometry analysis and then normalized with Smad3 or β-actin, respectively, as indicated. (F) NRK-49F cells were transfected control siRNA or JMJD siRNA and then incubated with medium containing 0.5% serum or treated with TGFβ1 (2 ng/ml) for 36 h. Cell lysates were prepared and subjected to immunoblot analysis with antibodies against pSmad3, Smad3, Smad7, β-actin. Expression levels of pSmad3 (G), Smad3 (H), Smad7 (I), DNMT1 (J) or β-actin were quantified by densitometry analysis and then normalized with Smad3 or β-actin as indicated in the figures. Values are the means ± sem of at ≥3 independent experiments. $^{**}P<0.01$

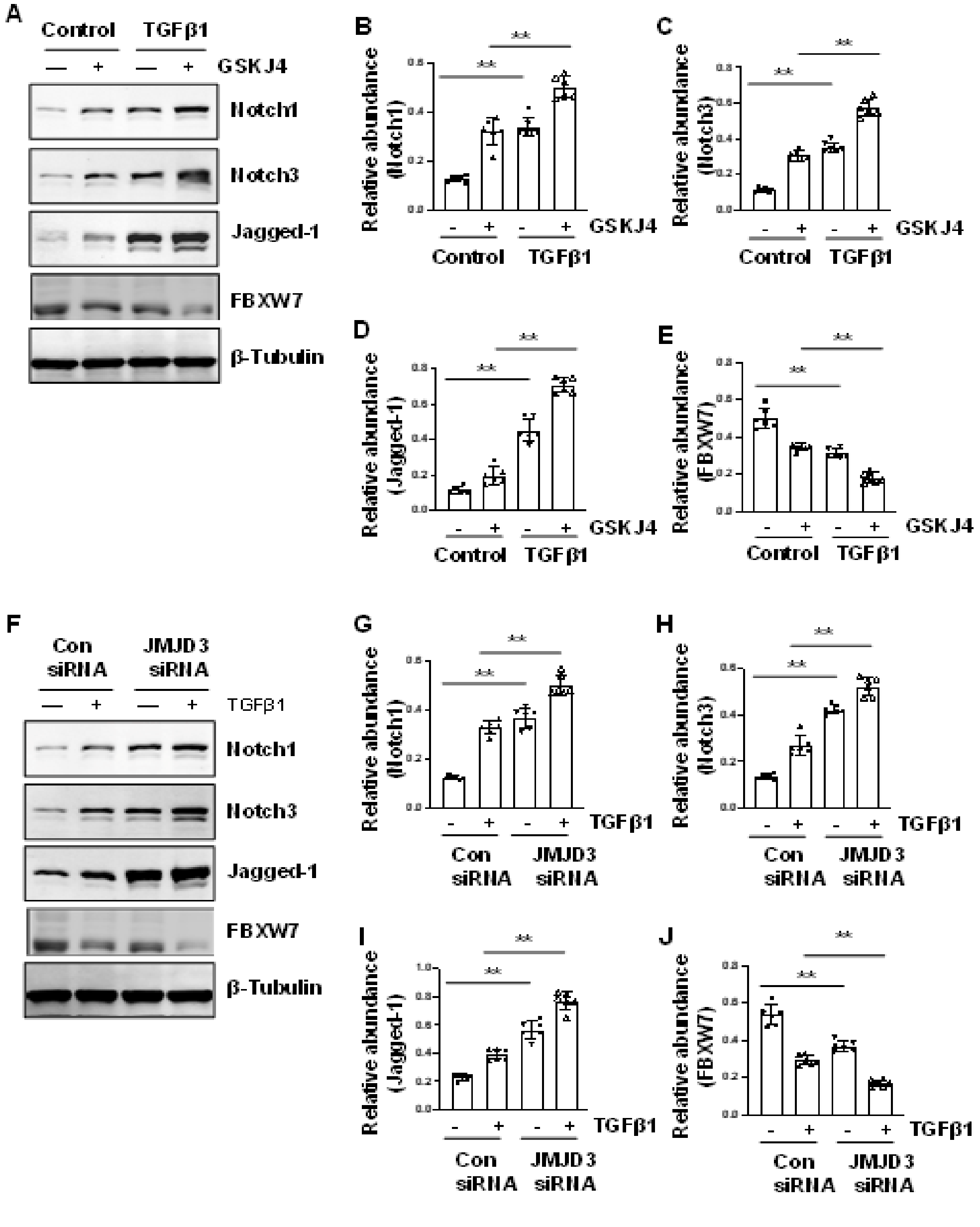

Figure S6. Inhibition of JMJD3 by GSKJ4 or siRNA enhances TGFβ1-induced expression of Notch1, Notch3 and Jagged-1 and reduced expression of FBXW7 in cultured mouse renal epithelial cells. (A) mTECS cells were incubated with medium containing 0.5% serum or treated with TGFβ1 (2 ng/ml) in the presence or absence of GSKJ4 (6 μM) for 36 h. Cell lysates were

prepared and subjected to immunoblot analysis with antibodies against Notch1, Notch3 and Jagged-1, FBXW7 or β-tubulin. Expression levels of Notch1 (B), Notch3 (C) and Jagged-1 (D), FBXW7 (E) or β-tubulin were quantified by densitometry analysis and then normalized with β-tubulin, respectively, as indicated in the figures. (F) mTECS cells were transfected control siRNA or JMJD siRNA and then incubated with medium containing 0.5% serum or treated with TGFβ1 (2 ng/ml) for 36 h. Cell lysates were prepared and subjected to immunoblot analysis with antibodies against Notch1, Notch3 and Jagged-1, β-tubulin. Expression levels of Notch1 (G), Notch3 (H) , Jagged-1 (I) or FBXW7 (J), β-tubulin were quantified by densitometry analysis and then normalized with β-tubulin, respectively, as indicated in the figures Values are the means ± sem of at ≥3 independent experiments. *$P<0.05$; **$P<0.01$
